# Incorporation of histone H3.1 suppresses the lineage potential of skeletal muscle

**DOI:** 10.1093/nar/gku1346

**Published:** 2014-12-24

**Authors:** Akihito Harada, Kazumitsu Maehara, Yuko Sato, Daijiro Konno, Taro Tachibana, Hiroshi Kimura, Yasuyuki Ohkawa

**Affiliations:** 1 Department of Advanced Medical Initiatives, Faculty of Medicine, Kyushu University, Fukuoka 812-8582, Japan; 2 Graduate School of Bioscience and Biotechnology, Tokyo Institute of Technology, Yokohama, 226-8501, Japan; 3 CREST, JST, 4-1-8 Honcho, Kawaguchi, Saitama, 332-0021, Japan; 4 Laboratory for Cell Asymmetry, Center for Developmental Biology, RIKEN, Kobe 650-0047, Japan; 5 Department Bioengineering, Graduate School of Engineering, Osaka City University, Osaka 558-8585, Japan

## Abstract

Lineage potential is triggered by lineage-specific transcription factors in association with changes in the chromatin structure. Histone H3.3 variant is thought to play an important role in the regulation of lineage-specific genes. To elucidate the function of H3.3 in myogenic differentiation, we forced the expression of GFP-H3.1 to alter the balance between H3.1 and H3.3 in mouse C2C12 cells that could be differentiated into myotubes. GFP-H3.1 replaced H3.3 in the regulatory regions of skeletal muscle (SKM) genes and induced a decrease of H3K4 trimethylation (H3K4me3) and increase of H3K27 trimethylation (H3K27me3). Similar results were obtained by H3.3 knockdown. In contrast, MyoD-dependent H3.3 incorporation into SKM genes in fibroblasts induced an increase of H3K4me3 and H3K27me3. In mouse embryos, a bivalent modification of H3K4me3 and H3K27me3 was formed on H3.3-incorporated SKM genes before embryonic skeletal muscle differentiation. These results suggest that lineage potential is established through a selective incorporation of specific H3 variants that governs the balance of histone modifications.

## INTRODUCTION

The development of multicellular organisms is accompanied by the acquisition of various differentiated cells. Cells acquire lineage potential toward specific directions during cell fate decision, and the lineage potential can be established by marking genes prior to their expression after differentiation. The expression of selected genes during differentiation is regulated by the structure of chromatin, which includes nucleosomes. Post-translational modifications of histones are regarded as signals for the compaction of chromatin and other protein complexes, acting as ‘on/off’ switches for the gene expression (1). One example is K4me3 in histone H3 (H3K4me3), which is localized around the transcription start sites (TSS) of actively transcribed genes. In contrast, K27me3 in histone H3 (H3K27me3) is associated with transcriptionally repressed chromatin. Even though these two modifications function antagonistically, their coexistence (known as bivalent modification) has been shown in many promoter regions of genes important for developmental lineage regulation in mouse embryonic stem (mES) cells (2–4). Therefore, H3K4me3 and H3K27me3 may mark lineage specific genes prior to their expression in differentiation.

The selective incorporation of the histone H3.3 variant is also involved in marking the genome for selective gene expression. H3.3 was reported to be incorporated in many transcriptionally active regions (5) and in lineage-specific genes in mES cells (6). H3.3 also plays a role in the inheritance of epigenetic memory in the nuclear transplant of *Xenopus* (7).

Several connections between individual histone modifications and variants have already been demonstrated. For example, H3K4me3 is more abundant in the H3.3 variant than in the major H3 variants (i.e. H3.1 and H3.2) incorporated into chromatin during replication (8–10). The H3.3-specific function of K27 has also been implicated *in vivo.* Mutations at K27 of *H3F3A* (which encodes H3.3) are associated with human pediatric glioblastoma (11) and are also known to cause abnormal heterochromatin formation in mouse embryos (12). In ES cells, distributions of H3.3 and the bivalent modification are correlated (6). These results suggest that H3.3 incorporation may provide a platform for specific modifications *in vivo*.

In myogenesis, both the selection of the histone variant and the appropriate modification play a critical role in selective gene expression. We previously reported that H3.3 is incorporated into skeletal muscle gene (SKM gene) loci prior to the gene expression (13). Bivalent modification is also found in C2C12 myoblasts (14), wherein the balance between H3K27me3 and H3K4me3 regulates the promotion of myogenesis. It has been shown that enhancer of Zeste homolog 2 (Ezh2), a H3K27 methyltransferase, plays a key role in K27 trimethylation on the regulatory region of SKM genes prior to differentiation (15–17). In addition, the elevation of H3K4me3 levels by Ash2L upon differentiation is coupled with the demethylation of H3K27me3 by UTX (18). These results suggest that myogenesis is controlled through sequential chromatin regulation by the selection of the histone variant and the appropriate histone modification. However, a direct connection between histone variant H3.3 and histone modification during differentiation has not been demonstrated. Here we examined the function of incorporated histone variants in myogenesis using C2C12 cells and mouse embryos. We found that selective H3.3 incorporation is essential for establishing specific modifications in SKM genes, suggesting that the incorporation of specific histone H3 variants determines the lineage potential of SKM (myogenic potential).

## MATERIALS AND METHODS

### Ethics statement

All animal procedures were conducted in accordance with the Guidelines for the Care and Use of Laboratory Animals and were approved by the Institutional Animal Care and Use Committee (IACUC) at Kyushu University.

### Cells

C2C12 cells or Chd2^WT^ and Chd2^KD^ cells generated previously (13) were grown in Dulbecco's modified Eagle's medium (DMEM) supplemented with 20% fetal bovine serum. Undifferentiated cells were harvested at 60–70% confluence. Differentiated cells were transferred to DMEM containing 2% horse serum upon reaching confluence (90–100%) and harvested 48 h later. The NIH3T3-derived cell (B22) line was infected with retrovirus expressing MyoD, as described previously (19).

### GFP-fused histone H3.1 variant expression constructs and cell line selection

*Hist1h3a* and *H3f3a* cDNA (purchased from Operon Biotechnologies) were used for the expression of H3.1 and H3.3. The cDNAs were ligated into the Bidirectional Tet Expression Vector pT2A-TRETIBI (modified Clontech Tet-On system), which contains TolII transposon elements and Enhanced Green Fluorescence Protein (EGFP) cDNA located upstream of the cDNA sequence, which was modified from pT2AL200R150G (20–22). Transfections of pT2A-TRETIBI/EGFP-H3.1, EGFP-H3.3, EGFP-H3.1 A31S and EGFP-H3.3 S31A were performed using Lipofectamine 2000 (Life Technologies, Carlsbad, CA, USA). C2C12 cells at 20–30% confluence were transfected with an expression vector (4 μg plasmid DNA per 100-mm plate), pCAGGS-TP coding transposase (provided by Dr Kawakami) and pT2A-CAG-rtTA2S-M2 and incubated for 24 h. To create cell lines stably expressing Green Fluorescence Protein (GFP)-fused histone H3 variants, transfected cells were cultured for 14–21 days in the presence of 1 μg/ml of doxycycline and 1 mg/ml of G418. Finally, GFP-positive cells were selected using fluorescence activating cell-sorting.

pT2A-TRETIBI/EGFP-H3.1 A31S and EGFP-H3.3 S31A were made from site-directed mutagenesis based on *Hist1h3a* and *H3f3a* cDNAs. Primers for the A31S and S31A mutations were as follows: sense and anti-sense primers for A31S, CAAGAGCGCCCCGTCCACCGGCGGCGTGAAG and CTTCACGCCGCCGGTGGACGGGGCGCTCTTG; sense and anti-sense primers for S31A, CAAGAGTGCGCCCGCTACTGGAGGGGTGAAG and CTTCACCCCTCCAGTAGCGGGCGCACTCTTG.

### FRAP

Fluorescence Recovery after Photbleaching (FRAP) was performed as described (23) using a confocal microscope (FV-1000; Olympus) with a 60× PlanApoN Oil SC NA = 1.4 lens. A confocal image of a ﬁeld containing 2–5 nuclei was collected (800 × 800 pixels, zoom 1.2, scan speed 2 μs/pixel, pinhole 800 μm, 4 line averaging, BA505 emission ﬁlter and 0.1% transmission of 488-nm Ar laser), one half of each nucleus was bleached using 100% transmission of a 488-nm laser and images were collected using the original setting at 5 min intervals.

### Immunocytochemistry

Cells were plated on cover slips, washed twice with phosphate buffered saline (PBS), fixed with 1% paraformaldehyde in PBS, permeabilized with 0.5% Triton X-100 in PBS and washed twice with PBS. A 15 min incubation with Blocking One (Nacalai Tesque Inc.) was followed by 2 h incubation with mouse anti-myogenin (F5D, Santa Cruz Biotechnology, 1:500; Figures 1B, 5B and E) or with rabbit anti-myosin heavy chain (Calbiochem, 1:100; Figure 1D) diluted with 10% Blocking One in PBS at room temperature. The coverslips were then washed three times with PBS and incubated for 30 min at room temperature with CF568-labeled goat anti-mouse or anti-rabbit antibody (1:1000; Biotium Inc.) and Bisbenzimide H33342 Fluorochrome Trihydrochloride (Hoechst) (1:5000; Nacalai Tesque Inc.) diluted with 10% Blocking One in PBS. Coverslips were again washed three times in PBS and mounted in ProLong Gold Antifade Reagent (Life Technologies). Images were visualized using a fluorescence microscope (BZ-9000; Keyence). Co-localization was evaluated using BZ-II Analyzer software (Keyence).

### Immunoblotting

Cells were washed twice with phosphate buffered saline (PBS), centrifuged, and then resuspended in 2x sodium dodecylsulphate (SDS) sample buffer. The samples were separated by SDS-PAGE (10–20% gel for histone variants and 5–20% gel for other proteins) and transferred to a nitrocellulose membrane or polyvinylidene fluoride membrane with Trans-Blot Turbo Transfer System (Bio-Rad Laboratories, Hercules and CA; 2.5 A, 25 V, 7 min). The membrane was blocked for 1 h in 5% (w/v) skim milk in Tris-buffered saline containing 0.05% (v/v) Tween 20 (TBST), then incubated with primary antibodies in Hikari Solution A (Nacalai Tesque Inc.) followed by incubation with horseradish peroxidase-labeled secondary antibodies and detection using the Chemi-Lumi One Ultra (Nacalai Tesque Inc.). The primary antibodies used included rabbit anti-Hsp90 (H-114, Santa Cruz Biotechnology, 1:1000; Supplementary Figures S1B and S5B), anti-H2B (Imagenex, 1:1000; Figure 2C, Supplementary Figures S1B and S3A-B), anti-H4 (Abcam, 1:1000; Supplementary Figure S1B), anti-H3K4me3 (07–473, Millipore, 1:2000; Supplementary Figures S3B and S3C), anti-H3K27me3 (07–449, Millipore, 1:2000; Supplementary Figures S3B and S3C), anti-Daxx (sc-7152, Santa Cruz Biotechnology, 1:1000; Supplementary Figure S4A), anti-Ezh2 (active motif, 1:2000; Figures 3B and C), mouse anti-GFP (GF200, Nacalai Tesque Inc., 1:500; Figures 2C, 3B-C, Supplementary Figures S1B, S3A-C and S5A-B), anti-Hira (WC119, Millipore, 1:1000; Supplementary Figure S4A), anti-H3.1/H3.2 (1D4F2, hybridoma supernatant, 1:1000; Supplementary Figure S5A) (13), rat anti-H3.3 (6C4A3, hybridoma supernatant, 1:1000; Figure 2C, Supplementary Figures S4A and S5A) (13), anti-H3.1/H3.2 (6G3C7, hybridoma supernatant, 1:1000; Figure 2C and Supplementary Figure S5A) (13) and anti-H3 (1G1, hybridoma supernatant, 1:1000; Supplementary Figures S1B, S3C, S4A and S5A-B) (24). Secondary antibodies were horseradish peroxidase-conjugated anti-rabbit, anti-mouse and anti-rat IgG antibodies (GE Healthcare, 1:5000).

**Figure 1.**
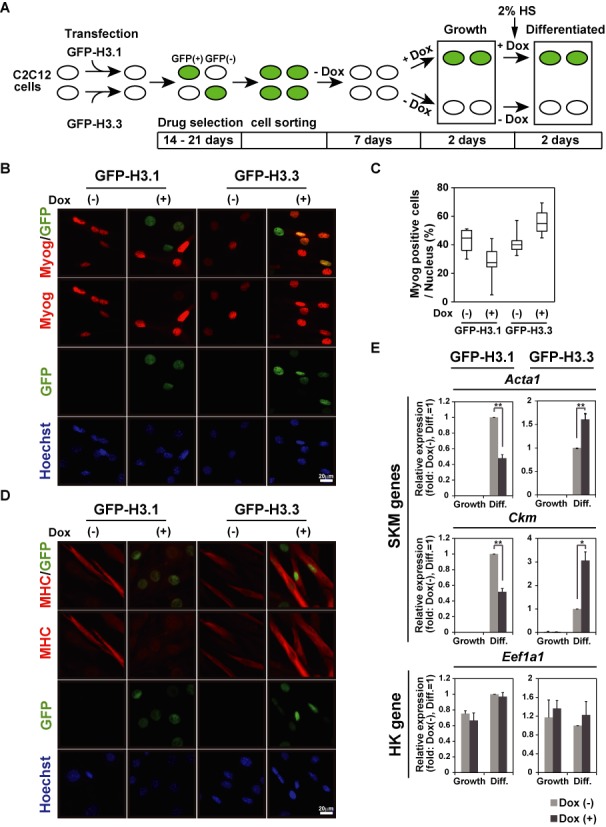
GFP-H3.1 overexpression, but not GFP-H3.3 overexpression, inhibits myogenesis. (A) Analysis scheme of GFP-expressing H3.1 and H3.3 variant cells. (B) Immunofluorescence with Myog. Myog expression was repressed in C2C12 cells when GFP-H3.1 was induced by Dox. Scale bars, 20 μm. (C) Box plot representation of Myog-positive fraction among total nuclei identified by Hoechst staining. Six different fields from images like (B) were analyzed. (D) Immunofluorescence with MHC. MHC expression was repressed in C2C12 cells after forced induction of GFP-H3.1 expression by Dox. Scale bars, 20 μm. (E) qPCR. mRNA expression levels of SKM marker genes were repressed in C2C12 cells expressing GFP-H3.1 (left panels), whereas GFP-H3.3 expression was not (right panels). Relative values to differentiated cells without Dox are presented (mean ± standard deviation of three independent experiments). *P < 0.05, **P < 0.01.

**Figure 2.**
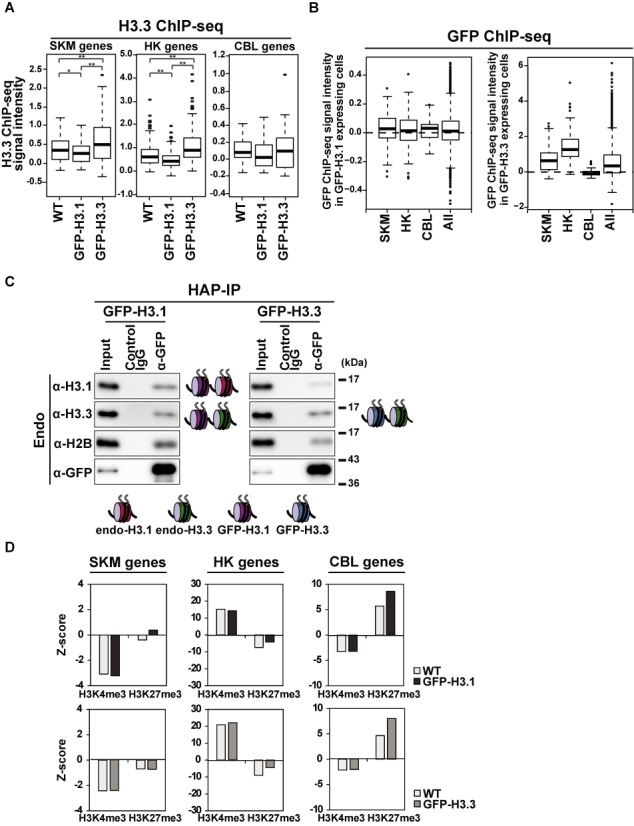
Global effects of GFP-H3.1 overexpression on endogenous H3.3 and modifications. (A) H3.3 enrichment in different gene sets. Box plots show H3.3 ChIP-seq signal intensities for the indicated gene sets at TSS ± 2 kb. Mann-Whitney test was used for the statistical analysis. *P < 0.05, **P < 0.01. Related data are shown in Supplementary Tables S4 and 5. (B) GFP-H3.1 or GFP-H3.3 incorporation in different gene sets expressed in box plots. GFP ChIP-seq signal intensities were analyzed at TSS ± 2 kb. Related data are shown in Supplementary Tables S4 and 5. (C) Co-localization of GFP-H3.1 and endogenous H3.3 in nucleosomes. Hydroxyapatite (HAP)-purified chromatin from C2C12 cells expressing GFP-H3.1 and GFP-H3.3 were immunoprecipitated with control IgG and GFP-specific antibody, and the presence of endogenous histones were analyzed by Western blotting. H3.3 was associated with GFP-H3.1-containing nucleosomes to form a bivariant state. (D) Association of H3K4me3 and H3K27m3 with different gene sets expressed as Z-scores from ChIP-seq signal intensities in each gene set at TSS ± 2 kb. GFP-H3.1 expression caused preferential H3K27me3 in SKM genes.

### Quantitative RT-PCR

Total RNA was isolated and reversed-transcribed with PrimeScript Reverse Transcriptase (Takara Bio Inc.) and an oligo dT primer, as previously described (13). qPCR was performed using Thunderbird qPCR Mix (Toyobo Co., Ltd.). Primers are listed in Supplementary Table S1. qPCR data were normalized to *Gapdh* expression levels and presented as the mean ± standard deviation of three independent experiments.

### Preparation of mouse embryonic tissue

Embryonic tissues were collected as described previously (25). For ChIP-Seq, the dissected tissue was homogenized using a Polytron Homogenizer (PT 10-35 GT; Kinematica AG, Switzerland) and washed twice with PBS-Mg+ and 1 × 10^7^ cells were fixed with 0.5% formaldehyde.

### Chromatin immunoprecipitation

ChIP assays were performed as described previously with some modifications (13). Cultured cells (1 × 10^7^ cells) were cross-linked in 0.5% formaldehyde and suspended in ChIP buffer (5 mM PIPES, 200 mM KCl, 1 mM CaCl_2_, 1.5 mM MgCl_2_, 5% sucrose, 0.5% NP-40 and protease inhibitor cocktail; Nacalai Tesque Inc.). The samples were incubated for 15 min on ice, sonicated for 5 s three times at 70% maximum amplitude with a Sonicator (VCX130; Sonics & Materials, Inc., CT, USA) and digested with micrococcal nuclease (1 μl; New England Biolabs, Ipswich, MA, USA) and Ribonuclease A (1 μl; Nacalai Tesque Inc.) at 37°C for 40 min. The digested samples were centrifuged at 15 000 × *g* for 10 min. Supernatant containing 4–8 μg DNA was incubated with a rat monoclonal antibody against H3.3 (1E4A3, hybridoma supernatant, 2 μg) (13) and mouse monoclonal antibodies against H3K4me3 (2 μg) (26), H3K27me3 (2 μg) (27), Ezh2 (Active motif, AC22, 2 μg) and GFP (Bio Academia Co. Ltd., 1A5, 2 μg) pre-bound to magnetic beads at 4°C overnight with rotation. The immune complexes were eluted from the beads using 1% SDS in TE, followed by washing with ChIP buffer and TE buffer (both twice). Cross-links were reversed with 0.5 M NaCl at 65°C for 4 h, treated with 2% proteinase K (Nacalai Tesque Inc. at 50°C for 1 h and DNA was purified by a Qiaquick polymerase chain reaction (PCR) purification kit (Qiagen, Valencia, CA, USA). In re-ChIP, elution were performed by re-ChIP elution buffer containing 1x TE, 2% SDS and 15 mM dithiothreitol (DTT) for 30 min at 37°C. The eluted samples were diluted 20 times in ChIP buffer and re-incubated with another antibody. Q-PCR analysis of immunoprecipitated DNA was performed using Thunderbird qPCR Mix (Toyobo Co., Ltd., Osaka, Japan). Relative enrichment was defined as the ratio of the quantity of amplified PCR product relative to 10% of the input genomic DNA. Quantification represents the mean of three independent experiments ± standard deviation. The primers used to amplify the promoter regions are listed in Supplementary Table S2.

### ChIP sequencing and read alignments

Sample preparation from GFP-fused histone H3.1 and H3.3 expressing cells and C2C12 wild-type (WT) cells or embryonic tissues was performed as described above. The ChIP library was prepared with the Illumina protocol and sequenced on Illumina HiSeq-1500 sequencers. The sequence reads for H3.3, H3K4me3, H3K27me3, GFP and Input were aligned to the reference mouse genome (mm9, build 37) using Bowtie 2 software (28). Unique tag numbers are listed in Supplementary Table S3.

**Figure 3.**
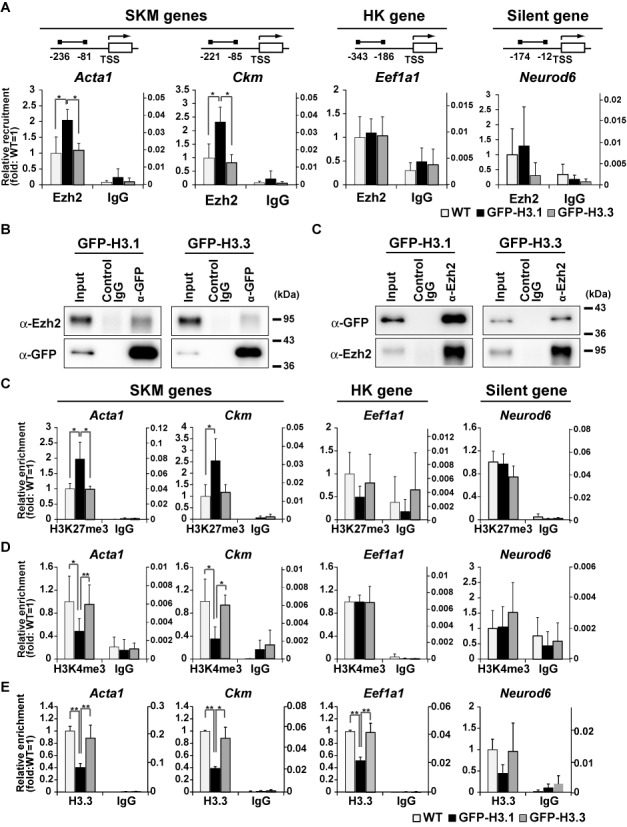
Increases in H3K27me3 level and H3.1 incorporation into SKM gene regulatory regions. (A) ChIP-qPCR assay using Ezh2-specific antibody. The regulatory regions of SKM, HK, and silent genes in GFP-H3.1 and GFP-H3.3 expressing cells and in WT cells at growth state were analyzed. Recovery efficiency (mean ± standard deviation of three independent experiments) is expressed as relative enrichment to WT cells (left axis) and ratio to input (right axis). The positions of PCR primers are indicated on top. *P < 0.05, **P < 0.01. (B and C) Association of GFP-H3.1 and Ezh2. Chromatin prepared from GFP-H3.1 expressing cells was immunoprecipitated and probed using antibodies directed against GFP (B) and Ezh2 (C). (D-F) ChIP-qPCR assays. H3K27me3 (D), H3K4me3 (E), and H3.3 (F) were analyzed as in (A). Recovery efficiency (mean ± standard deviation of three independent experiments) is expressed as relative enrichment to WT cells (left axis) and ratio to input (right axis). *P < 0.05, **P < 0.01.

### Data analysis

To analyze the data from ChIP-seq, we counted the number of ChIP-seq unique tags in each window (for each figure) and normalized as reads per kb per million (normalized for each sample according to the total number of unique tags). Signal intensities (*SI*) are defined as follows:

\begin{eqnarray*}
&&SI = (RPKM \text{ of the unique Tag count for the IPed sample })- \nonumber \\
&&\quad \quad \quad \quad \quad (RPKM \text{ of the unique tag count for the input})
\end{eqnarray*}

**Figure 4.**
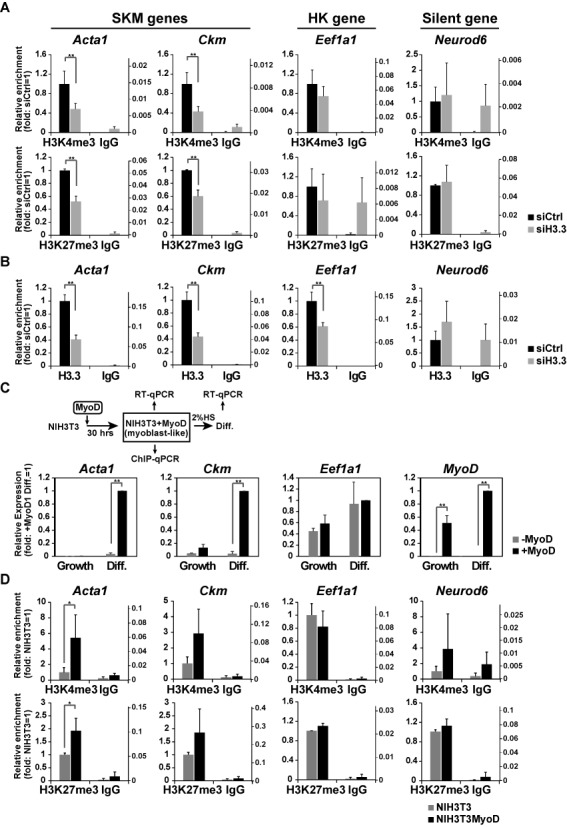
Bivalent modifications at SKM-gene regulatory sequences are dependent on H3.3. ChIP-qPCR assays were performed as in Figure3A. (A and B) H3K4me3 enrichment (A, upper panels), H3K27me3 enrichment (A, bottom panels), and H3.3 (B) were analyzed using C2C12 cells treated with either control siRNA or H3.3 siRNA. Recovery efficiency (mean ± standard deviation of three independent experiments) is expressed as relative enrichment to WT cells (left axis) and ratio to input (right axis). *P < 0.05, **P < 0.01. (C and D) Effects of MyoD-induced H3.3 incorporation into SKM genes on mRNA expression and histone modifications. The experimental scheme is illustrated (C, top). (C) mRNA levels analyzed by qPCR. Relative values to differentiated cells expressing NIH3T3+MyoD are shown (mean ± standard deviation of three independent experiments). (D) ChIP assays. Recovery efficiency (mean ± standard deviation of three independent experiments) is expressed as relative enrichment to WT cells (left axis) and ratio to input (right axis). *P < 0.05, **P < 0.01.

Each ChIP-seq *SI* was calculated using 200 bp windows (Figure 6A, Supplementary Figures S2A and S6B), 2 kb windows (Supplementary Figure S6A) or windows around ± 2 kb a TSS (Figures 2A-B, D and 6B). This definition of ChIP-seq *SI* was also used by (29).

**Figure 5.**
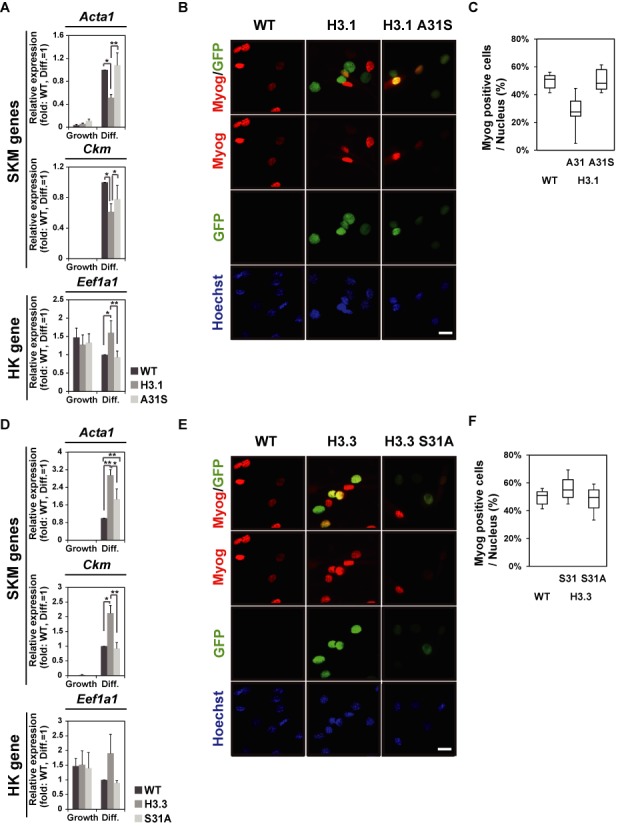
GFP-H3.1 A31S overexpression does not inhibit myogenesis and GFP-H3.3 S31A overexpression does not enhance myogenesis. (A) Q-PCR. mRNA expression levels of SKM marker genes were repressed in C2C12 cells expressing GFP-H3.1, whereas they were not in those expressing GFP-H3.1 A31S. Relative values to differentiated WT cells are presented (mean ± standard deviation of three independent experiments). (B) Immunofluorescence with Myog. (C) Box plot representation of the Myog-positive fraction among total nuclei identified by Hoechst staining. Ten different fields from images like (B) were analyzed. Scale bar, 20 μm. (D) Q-PCR. mRNA expression levels of SKM marker genes were enhanced in C2C12 cells expressing GFP-H3.3, whereas it was not in those expressing GFP-H3.3 S31A. Relative values to differentiated WT cells are presented (mean ± standard deviation of three independent experiments). (E) Immunofluorescence with Myog. (F) Box plot representation of the Myog-positive fraction among total nuclei identified by Hoechst staining. Ten different fields from images like (E) were analyzed. Scale bar, 20 μm.

The correlation matrix was calculated based on the *SI* in a 2 kb scale for the whole genome. Mean correlation values show the correlation between three biological replicates.

To estimate the effect of the observed histone modification state affected by the expression of the histone H3 variant, we used a *Z*-score (standard deviations from the global average). Each *Z*-score was computed for each gene with GFP-incorporation intensity signals. Histone modification densities were calculated in windows around ± 2 kb a TSS. These data are listed in Supplementary Tables S4 and S5.

To visualize ChIP-seq data with Integrative Genomics Viewer (IGV; version 2.3), *SI* (signal height in IGV) was calculated in 2 kb windows using 1 kb intervals along the mouse genome, i.e. *SI*s with 50% overlapping 1 kb windows. Forelimb- and hindlimb-specific genes were selected from a previous report (30).

For bivalent modification analysis, the relative ratio of H3K4me3 and H3K27me3 histone modifications was expressed as the angle,

\begin{equation*} \varphi = 2\arctan [ + \{ (SI)_{{\rm H}3{\rm K}27{\rm me}3} \} / + \{ (SI)_{{\rm H}3{\rm K}4{\rm me}3} \} ] \end{equation*}

*SI*s of both H3K4me3 and H3K27me3 were calculated in ± 2 kb windows within a TSS of each tissue-specific gene. φ ranges from 0 to 180° (0° corresponds to only H3K4me3 and 180° corresponds to only H3K27me3). To assume a negative *SI* as no-signal, we filtered *SI* using the function +(x) = x when x has positive value or +(x) = 0 when x has negative value. In the case where *SI* of H3K4me3 has no signal and the *SI* of H3K27me3 is positive, i.e. +{(*SI*)_H3K4me3_} = 0 and +{(*SI*)_H3K27me3_} > 0, then φ = 180°. The condition of both *SI*s of H3K4me3 and H3K27me3 having no-signal was excluded from our analysis.

Tissue-specific genes were selected from the tissue-specific genes database (http://bioinf.xmu.edu.cn:8080/databases/TiSGeD/index.html) (31). SKM genes were selected from genes with specification measure (SPM) specificity parameters of 0.7–1.0 (the nearer the SPM is to 1, the higher the specificity) and with a fragments per kb of exons per million fragments mapped (FPKM) value <100 in proliferating cells or a FPKM value at least threefold higher than that of proliferating cells in differentiated cells, according to C2C12 mRNA-seq. cerebellum (CBL) genes were selected from genes with a SPM of 0.7–1.0 and a FPKM value that was <0.5 in C2C12 mRNA-seq. Because HK genes are not tissue-specific, we used the same genes as those from our previous study (13). These data are listed in Supplementary Tables S5–S8.

### RNA-Seq library preparation and analysis (RNA-Seq)

Total RNA at the growth and differentiated (i.e. post-differentiation) states for C2C12 cells were obtained as described above. Library preparation and sequence analysis were performed by a protocol described previously (32).

### Immunoprecipitation

Immunoprecipitation (IP) was performed as described (13). Cleared lysates were rocked with 4 μg of monoclonal antibody against Ezh2 (AC22, Active motif) and GFP (Bio Academia Co. Ltd., 1A5) for 12 h.

### Immunoprecipitation using hydroxyapatite chromatography

Analysis of nucleosome levels was performed using samples purified by hydroxyapatite after the nuclear soluble fractions were removed, as described previously (23,33). Cells were washed twice with PBS and then lysed in ice-cold physiological buffer (PB; 100 mM CH_3_COOK, 30 mM KCl, 10 mM Na_2_HPO_4_, 1 mM DTT, 1 mM MgCl_2_, 1 mM ATP, 0.1% Triton X-100 and protease inhibitor cocktail; Nacalai Tesque Inc.). Lysates were incubated for 10 min on ice and then centrifuged at 1000 × *g* for 5 min. The pellets were resuspended in IP buffer (5 mM PIPES, 200 mM KCl, 1 mM CaCl_2_, 1.5 mM MgCl_2_, 5% sucrose, 0.5% NP-40 and protease inhibitor cocktail; Nacalai Tesque Inc.). The samples were sonicated for 1 s three times and digested to lengths corresponding to mono-, di- and tri-nucleosomes with micrococcal nuclease (1 μl; New England Biolabs) and Ribonuclease A (1 μl; Nacalai Tesque Inc.) at 37°C for 30 min. To stop the digestion reaction, ethylenediaminetetraacetic acid (EDTA) was added to the samples at a final concentration of 5 mM. 5M NaCl was then added to achieve a concentration of 0.5 M and the samples were transferred to a spin column including hydroxyapatite resin (100 mg; Nacalai Tesque Inc.) pre-washed with hydroxyapatite buffer 1 (HAPB1; 5 mM NaPO_4_, 600 mM KCl and 1 mM EDTA). The chromatin/hydroxyapatite mixture was incubated on a rotator for 30 min at 4°C and washed twice with HAP1B and hydroxyapatite buffer 2 (HAPB2; 5 mM NaPO_4_, 100 mM KCl and 1 mM EDTA). The purified nucleosomes were eluted with hydroxyapatite (HAP) elution buffer (500 mM NaPO_4_, 100 mM KCl and 1 mM EDTA). The nucleosomes were diluted 10 fold with IP buffer and rocked with 4 μg of rat monoclonal antibody against GFP (Bio Academia Co. Ltd., 1A5) or mouse monoclonal antibody against H3K4me3 (26) and H3K27me3 (27) and bound to rabbit anti-mouse IgG (Jackson ImmunoResearch Laboratories, West Grove, PA, USA) and anti-rabbit IgG magnetic beads (Veritas, Tokyo, Japan) for 12 h. Beads were washed three times in IP buffer and eluted with 2x SDS sample buffer.

### siRNA-mediated knockdown of MyoD, Hira, Daxx and H3.3

Knockdown of *MyoD* was performed as described previously (13). siRNA for *Hira, Daxx* and H3.3 (*H3f3a* and *H3f3b*) were purchased from Dharmacon: *Hira* (ON-TARGETplus SMARTpool #L-043074), *Daxx* (ON-TARGETplus SMARTpool #L-044803), *H3f3a* (ON-TARGETplus SMARTpool #L-042679), *H3f3b* (ON-TARGETplus SMARTpool #L-042835) and non-targeting (ON-TARGETplus SMARTpool #D-001810).

### Statistical test

Statistical significance in RT-qPCR and ChIP-qPCR data were evaluated using the Welch *t*-test.

## RESULTS

### Effect of H3.1 overexpression on myogenic differentiation

We previously showed that H3.3 is incorporated into myogenic genes prior to differentiation (13). To address the specific role of H3.3 in differentiation, we forced H3.1 expression to replace H3.3 in chromatin regions with high H3 turnover. For this purpose, we established Tet-On-C2C12 cells that harbor the N-terminal-GFP-H3.1 gene under a doxycycline (Dox)-inducible promoter. In the presence of Dox for 48 h, ∼68% of cells became GFP-positive during the growth (Supplementary Figure S1A). To evaluate the specific role of H3.1, GFP-H3.3 expressing cells were also established and used as controls. Dox-dependent expression of GFP-H3.1 or GFP-H3.3 was confirmed by immunoblotting using whole cell lysate (Supplementary Figure S1B) and their incorporation into chromatin was supported by the association with HAP-purified nucleosomes (Supplementary Figure S1C; nucleosomal GFP-H3.1 and GFP-H3.3 were estimated to occupy ∼20 and ∼12% of the total H3, respectively) and by little fluorescence recovery after photobleaching in living cells (Supplementary Figure S1D).

The myogenic potential in GFP-H3.1 expressing cells were evaluated by immunostaining with myogenic markers, myogenin (Myog) and myosin heavy chain (MHC), after the induction of differentiation in the absence (Dox-) or presence of Dox (Dox+) for two days (Figure 1A). Myog was expressed in a smaller number of GFP-H3.1-expressing (Dox+) cells than in control Dox- cells (25 versus 45%) (Figures 1B and C). MHC-positive myotubes were also less frequently observed in GFP-H3.1-expressing cells (Figure 1D). Furthermore, quantitative RT-PCR revealed that mRNA expression levels of SKM genes (i.e. *Acta1* and *Ckm*) were lower in cells expressing GFP-H3.1 compared to Dox- cells after differentiation was induced (Figure 1E). In contrast, Myog-positive cells were more present in Dox+ GFP-H3.3-expressing cells than Dox- cells (55 versus 40%) (Figure 1B-C). The proportion of MHC-positive myotubes in GFP-H3.3-expressing cells was slightly higher than that in Dox- cells (Figure 1D) and the mRNA expression levels of SKM genes were increased (Figure 1E). The expression level of a housekeeping (HK) gene, *Eef1a1*, was similar in these cells. In control C2C12 cells that harbored the Dox-inducible GFP gene, Dox treatment had no effect on SKM gene expression (Supplementary Figure S1E). These data indicate that the forced expression of H3.1 had an inhibitory effect on myogenic potential.

### Exogenously expressed GFP-H3.1 replaced H3.3 in SKM gene promoters

Since H3.3 is incorporated into the promoter regions of SKM genes before differentiation (13), we considered whether the exogenous expression of GFP-H3.1 could replace endogenous H3.3 at critical regulatory regions. To investigate chromatin incorporation of GFP-H3.1 and its impact on the endogenous H3.3 distribution, we performed chromatin IP combined with deep sequencing (ChIP-seq) using specific antibodies directed against H3.3, which has been shown to be highly specific to H3.3 over H3.1 (13). We analyzed the enrichment of H3.3 in growing cells (before inducing differentiation) at the promoter regions (± 2 kb of the TSSs) (13,34) in three gene subsets [i.e. 59 SKM genes, 150 HK genes and. 31 silenced CBL genes]. H3.3 was slightly less incorporated into SKM and HK genes in GFP-H3.1 expressing cells than in WT cells (Figure 2A), whereas the levels of H3.3 incorporation in both SKM and HK genes were high in GFP-H3.3-expressing cells. This result suggests that GFP-H3.1 replaced H3.3 in regions where frequent histone-exchange occurred, even though most GFP-H3.1 is likely to be incorporated into chromatin evenly by a replication-coupled manner (Figure 2B). Little H3.3 was incorporated in CBL genes of all three cell types, which is consistent with a previous report that found H3.3 was less incorporated in the loci of silent genes (35). The H3.3 distribution in the gene body was more enriched in the HK gene than in SKM or CBL genes (Supplementary Figure S2A), consistent with a previous report (36). In summary, the incorporation of H3.3 in GFP-H3.1 expressing cells was inhibited around TSSs in SKM genes and both TSSs and gene bodies in HK genes.

To investigate whether GFP-H3.1 is incorporated into nucleosomes surrounding those containing H3.3, we performed ChIP-western analysis. Chromatin fractions prepared from GFP-H3.1 were treated with micrococcal nuclease to release mono- to tri-nucleosomes, which were then purified with HAP (Supplementary Figure S2B). IP of HAP-purified nucleosomes (HAP-IP) with anti-GFP antibodies was then performed to determine which H3 variants were present in nucleosomes containing GFP-H3.1. Western blotting using specific antibodies directed against H3.1 (which reacts with H3.1 and H3.2) and H3.3 showed that H3.1/H3.2 and H3.3 were associated with GFP-H3.1, whereas H3.3 was preferentially associated with GFP-H3.3 (Figure 2C). These data support the view that overexpressed GFP-H3.1 is incorporated into the chromatin region that contained H3.3, resulting in a ‘bivariant’ state (a mixture of H3.1/H3.2 and H3.3 nucleosomes) in GFP-H3.1-expressing cells. Additionally, HAP-IP in GFP-H3.3-expressing cells showed less H2B in the fraction compared with GFP-H3.1-expressing cells, suggesting that the number of GFP-H3.3 nucleosomes is less than GFP-H3.1 nucleosomes. This result is consistent with previous reports that showed nucleosomes containing H3.3 have a relatively short turnover and are easy to be evicted from the genome (37–39).

### Replacing H3.3 with GFP-H3.1 shifts modification toward H3K27me3 in SKM genes

Since the overexpression of GFP-H3.1 converts H3.3-enriched regions to bivariant regions in muscle genes, we hypothesized that the bivariant state might associate with altered histone modifications. We focused on H3K4me3 and H3K27me3 because of their critical role in myogenesis (18). To evaluate the degree of H3K4me3 and H3K27me3 and bias toward either modification, we calculated the ChIP-seq signal difference between the average of a gene set and the average of all genes divided by the standard error (*Z*-score). In GFP-H3.1 expressing cells, SKM genes showed higher H3K27me3 levels than the average of all genes (*Z* = 0), while in WT cells, the H3K27me3 level was lower (Figure 2D).

To examine whether H3K4me3 and H3K27me3 modifications occurred on the nucleosomes containing GFP-H3.1, we conducted HAP-IP using antibodies directed against H3K4me3 and H3K27me3. Immunoblotting confirmed that H3K4me3 and H3K27me3 modifications in endogenous H3 (Supplementary Figure S3A). Nucleosomes containing GFP-H3.3 were prepared as controls. Modifications of H3K4me3 and H3K27me3 on H3 were also found by reciprocal HAP-IP using anti-GFP and immunoblotting with modification-specific antibodies (Supplementary Figure S3B). H3K4me3 had similar levels of GFP-H3.1 and GFP-H3.3 while H3K27me3 levels were higher in GFP-H3.1. Furthermore, GFP-H3.1-expressing cells contained nucleosomes that were modified by H3K27me3 preferentially (Supplementary Figure S3C). These data suggest that the selective formation of the bivariant state on SKM gene loci at growth state caused an increase in H3K27me3 and decrease in H3K4me3 levels. As a result, SKM gene loci may have lost their lineage potential and no longer expressed SKM genes during differentiation.

To address the mechanism that allows H3.1 to preferentially induce H3K27me3 in SKM genes, we assessed the involvement of Ezh2, an enzyme that catalyzes the modification of H3K27me3. Ezh2 has been reported to be recruited to myogenic genes prior to C2C12 cell differentiation (18) and also been suggested to associate with H3.1 (40). ChIP-qPCR revealed that at growth state, Ezh2 recruitment is increased in SKM gene loci (*Acta1* and *Ckm*) in GFP-H3.1-expressing cells compared with WT cells, but unchanged in either HK (*Eef1a1*) or silent (*Neurod6*) gene loci (Figure 3A). We further observed association between Ezh2 and exogenously expressed GFP-H3.1 and GFP-H3.3 by reciprocal co-IP using anti-GFP and anti-Ezh2 antibodies (Figures 3B and C). These data suggest that the incorporation of GFP-H3.1 enhances the recruitment of Ezh2 to enrich H3K27me3 on SKM genes. We further evaluated H3K4me3 and H3K27me3 levels in the regulatory regions of SKM genes by ChIP-qPCR assays. In GFP-H3.1 cells, H3K4me3 and H3K27me3 levels in *Acta1* and *Ckm* were higher and lower, respectively, than those in WT cells, while their levels were little changed in *Eef1a1* and *Neurod6* (Figures 3D and E). In contrast, H3K4me3 and H3K27me3 levels in all genes were the same in GFP-H3.3-expressing cells and WT cells. ChIP-qPCR using H3.3 specific antibody revealed that H3.3 was less incorporated into the same regulatory regions of SKM genes (*Acta1* and *Ckm*) and a HK gene (*Eef1a1*) in GFP-H3.1 expressing cells at growth state (Figure 3F). These data are consistent with the view that GFP-H3.1 replaces H3.3 in the regulatory regions of SKM genes and then alters the balance of modifications toward more H3K27me3. The active transcription of HK genes may have contributed to the maintenance of H3K4me3 levels even when the H3.3 level was reduced (34).

### The inhibition of H3.3 incorporation by knockdown diminished the occurrence of both H3K4me3 and H3K27me3 modifications in the regulatory regions of SKM genes

To verify the specific role of H3.3 incorporation, we inhibited H3.3 incorporation by knockdown of a histone H3.3 chaperone as well H3.3 itself (Supplementary Figure S4A). Hira has been reported to mediate H3.3 incorporation into chromatin in various cells including C2C12 cells (34,40–43). Daxx is reported to be another H3.3 chaperone with a more specific role in telomeres and pericentromeric regions or Promyelocytic Leukemia (PML) bodies (6,44,45). The expression of *Acta*1, *Ckm* and *MyoD* in differentiated C2C12 cells was lowered by knockdown of H3.3 and Hira, but not knockdown of Daxx (Supplementary Figure S4B) (40). This result suggests that Hira, rather than Daxx, plays a major role in H3.3 incorporation into SKM genes. Because Hira knockdown inhibited *MyoD* expression at growth state (Supplementary Figure S4B), which can affect the expression of downstream SKM genes, we used H3.3 knockdown for further analysis.

In H3.3-knockdown (H3.3 KD) cells at growth state, the levels of H3K4me3 and H3K27me3 in SKM genes were significantly decreased compared to control cells (Figure 4A). In contrast, the levels of H3K4me3 and H3K27me3 in *Eef1a1* and *Neurod6* did not change significantly. H3.3 was less incorporated in the HK and SKM genes in H3.3 KD cells (Figure 4B). These results are consistent with GFP-H3.1 overexpression data. To further confirm the role of H3.3 on SKM genes, we also knocked down either Chd2, which mediates H3.3 incorporation into SKM genes or MyoD, which is the transcription factor that recruits the Chd2/H3.3 complex to the SKM gene loci (13). It has been shown that H3.3 incorporation is lowered specifically to SKM gene loci under these knockdown conditions (13). In WT cells, H3K4me3 levels in SKM genes (*Acta1* and *Ckm*) were increased during differentiation (Supplementary Figure S4C). In Chd2 KD cells, both H3K4me3 and H3K27me3 levels in SKM genes were lower than in WT cells at growth state and remained unchanged after differentiation. Chd2 KD did not affect the levels of H3K4me3 in the HK gene (*Eef1a1*) or H3K27me3 in the CBL gene (*Neurod6*), consistent with locus-specific H3.3 incorporation mediated by Chd2. Similar results were obtained by MyoD KD (Supplementary Figure S4D). Thus, the inhibition of H3.3 incorporation affected both H3K4me3 and H3K27me3, a phenotype more severe than that observed by GFP-H3.1 overexpression, which only enhanced H3K27me3.

**Figure 6.**
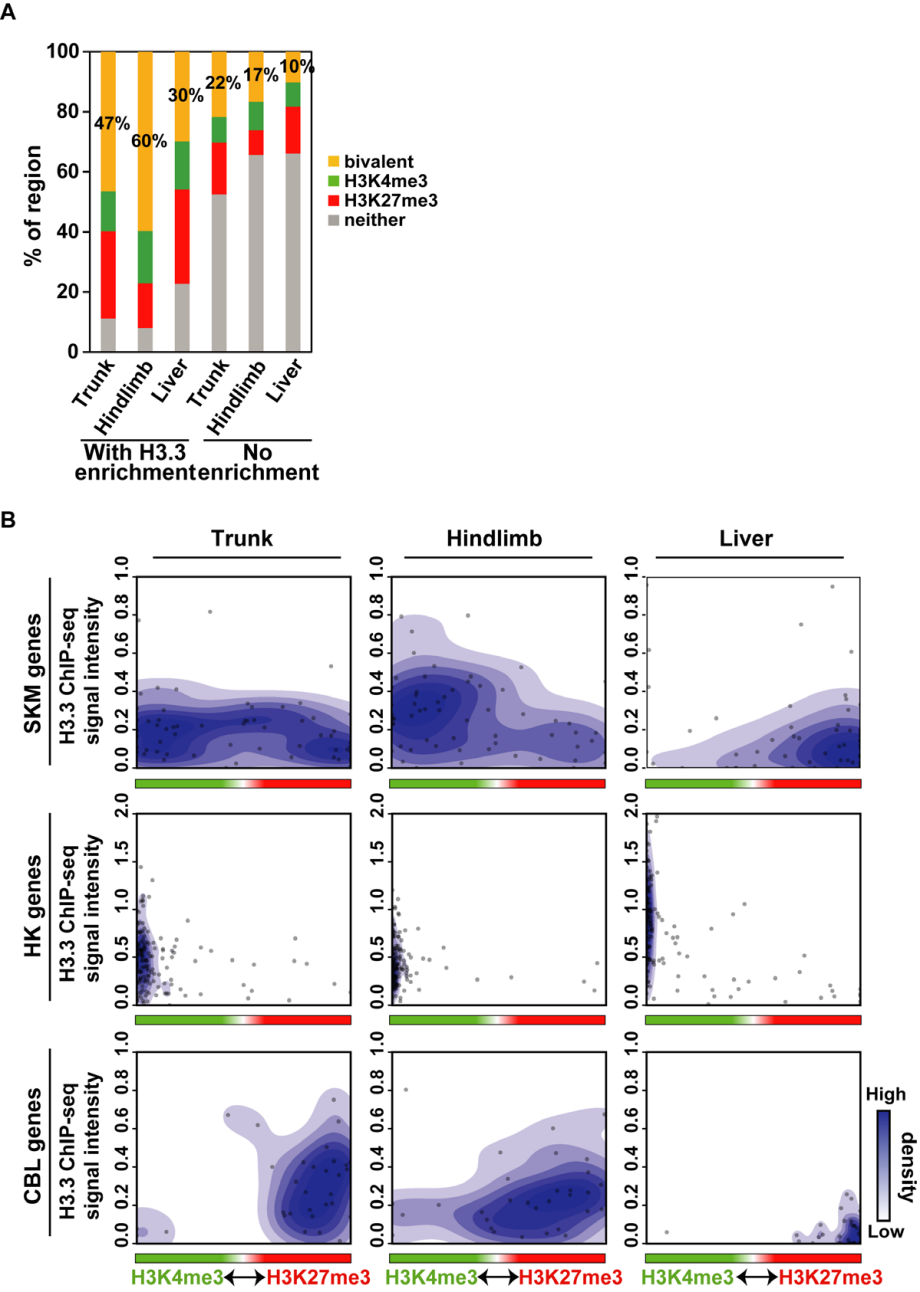
Bivalenthistone modifications form on H3.3-containing SKM gene loci in mouse embryo. (A) H3.3-enriched regions showed more bivalent modifications than those exhibiting no modifications and tended to colocalize with H3K4me3 and H3K27me3 modifications. (B) The quantitatively balanced state of the bivalent modification was associated with H3.3 incorporation. The scatter plot represents the relative ratio of H3K4me3 and H3K27me3 modifications (x-axis) and H3.3 ChIP-seq signal intensities (y-axis) in tissue-specific genes at TSS ± 2 kb. Related data are shown in Supplementary Tables S6-8.

To further analyze the impact of H3.3 on histone modifications, we induced H3.3 incorporation into SKM genes in NIH3T3 cells, where SKM genes were not competent for activation like C2C12 cells, by ectopic expression of MyoD (13,46). Ectopic expression of MyoD induced the expression of myogenic genes (*Acta1* and *Ckm*) under differentiation conditions (Figure 4C). In NIH3T3 + MyoD cells, both H3K4me3 and H3K27me3 levels in SKM genes were increased compared with NIH3T3 cells at growth state (Figure 4D). In contrast, modification levels in non-SKM genes remained unchanged (Figure 4D). These results are consistent with the association of bivalent modification with H3.3 incorporation. Taken together, in myogenic model systems based on cultured cells, H3.3 incorporation into myogenic genes can induce both H3K4me3 and H3K27me3 to establish lineage potential, while H3.1/H3.2 is preferentially associated with H3K27me3 for gene silencing (Supplementary Figure S4E).

To further address the functional difference between H3.1 and H3.3 at the molecular level, we examined the amino acid residues required for myogenic potential. We established cells expressing mutant GFP-H3.1, in which alanine 31 was substituted to serine (H3.1 A31S) or mutant H3.3, in which serine 31 was substituted to alanine (H3.3 S31A). Analysis using HAP-purified nucleosomes revealed that GFP-H3.1 A31S and GFP-H3.3 S31A were incorporated, perhaps by specific chaperones (47–49), at the same level as GFP-H3.1 and GFP-H3.3, respectively (Supplementary Figure S5). Quantitative RT-PCR and immunostaining of Myog unveiled that GFP-H3.1 A31S-expressing cells did not inhibit myogenic differentiation, which is unlike GFP-H3.1 (Figures 5A, B and C). Conversely, GFP-H3.3 S31A-expressing cells did not enhance differentiation (Figures 5D E and F), which is unlike GFP-H3.3. These data suggest that specific amino acid 31 is required for the function of each H3 variant.

### Bivalent modifications on H3.3 are enriched in SKM genes before the onset of embryonic gene expression

To investigate H3.3 incorporation into differentiation genes during embryogenesis *in vivo*, we performed ChIP-seq for H3.3, H3K4me3 and H3K27me3 using mouse embryonic tissues. We used the trunk at E10.5 (where myogenic genes are committed but not expressed), the hindlimb (myogenic genes are expressed) and liver (myogenic genes are silent) at E14.5 (25). Because the embryonic tissue samples were quantitatively very limited, which may jeopardize ChIP-seq quality, we performed ChIP-seq in triplicate to confirm reproducibility (Supplementary Figure S6A). The average correlation coefficient values for H3.3 were high (>0.6), as were those for H3K4me3 and H3K27me3 (>0.5), indicating that the ChIP-seq data of embryonic tissue had good correlation. Therefore, the average value of the three datasets was used for further analyses.

We first analyzed the enrichments of H3.3 in gene sets representative of HK, SKM and silent genes as described previously (13). In the trunk, H3.3 was particularly enriched in the promoter region of SKM genes, while in the hindlimb enrichment was spread along the gene body (Supplementary Figure S6B). As expected, H3.3 was not enriched in SKM genes in the liver where SKM genes are not expressed. Also, HK gene loci showed no remarkable differences among these tissues, with most enrichment observed at the transcription initiation and termination sites, as reported previously (36). In all tissues, silent genes showed no enrichment of H3.3.

Next, we focused on gene sets specifically expressed in the hindlimb or forelimb at E14.5 (30) to assess the occurrence of H3K4me3 and H3K27me3 modification on H3.3-incorporated regions. In the trunk at E10.5, when the hindlimb and forelimb are not yet formed, H3K4me3 and H3K27me3 were detected around the TSS of genes expressed at E14.5 in the hindlimb (*Hoxc10, Pitx1, Tbx4* and *Dmrt2*) and forelimb (*Hoxb6, Hoxb5, Hoxc5* and *Tbx5*) (Supplementary Figure S6C). H3.3 was also present at the TSS of these genes in both tissues. H3K4me3 in hindlimb genes became more abundant in E14.5 hindlimb tissue than that in E10.5 trunk tissue. In contrast, H3K4me3 and H3K27me3 were decreased, albeit slightly, in forelimb-specific genes. In the liver, H3K27me3 was highly observed in both gene groups and little H3.3 and H3K4me3 were detected.

We also analyzed the correlation of the bivalent state and H3.3 incorporation. For an overview of the colocalization, every 200 bp window (close to the mono-nucleosome size) over the entire genome was binarized into ‘enriched’ or ‘depleted’ based on the ratio of the ChIP signal to input. If the ChIP/input ratio in a region was >0 or <0, the region was scored as ‘enriched’ or ‘depleted’, respectively. In this analysis, 63, 74 and 70% of the genome in the trunk, hindlimb and liver, respectively, was scored as H3.3 enriched. We then determined whether either or both H3K4me3 and H3K27me3 were also enriched on H3.3-enriched or -depleted regions, finding the bivalent state in H3.3-enriched regions was 47, 60 and 30% in the trunk, hindlimb and liver, respectively, which was substantially higher than values in H3.3-depleted regions (22, 17 and 10%) (Figure 6A). These results indicated that bivalent modification occurs preferentially in H3.3-enriched regions, which is consistent with a previous report showing that H3.3 receives H3K4me3 and H3K27me3 modifications more easily than H3.1 (9). Curiously, the bivalent modification was more frequently observed in the hindlimb than in the trunk, although the hindlimb is more differentiated and the bivalent state is supposed to be diminished. The reason for this observation is currently unknown and requires further investigation. In fact, the H3.3 positive fraction was also higher in the hindlimb than trunk (74 versus 63%), which may suggest a more heterogeneous population in the hindlimb. Regardless, H3.3-containing regions are more associated with bivalent modification in all tissues.

**Figure 7.**
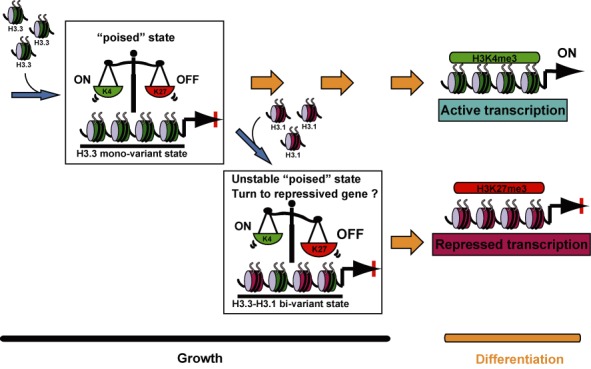
A model for lineage regulation by histone variants and the balance of bivalent modifications. H3.3 is incorporated into lineage-specific genes to stabilize the quantitative balance of bivalent modifications and maintain genes in the poised state. Incorporation of H3.1 into lineage-specific genes shifts the quantitative balance of bivalent modifications toward repression (i.e. heavy modification of H3K27me3), resulting in a loss of multi-lineage potential.

To examine the relationship between H3.3, H3K4me3 and H3K27me3 modifications in detail, ChIP-seq signals within ± 2 kb of the TSS of each set of genes (SKM, HK and CBL) were evaluated quantitatively. The H3.3 signal intensity was plotted against the relative ratio of H3K4me3 and H3K27me3 levels in each gene (Figure 6B). In the E10.5 trunk, H3.3 was incorporated into SKM genes with bivalent modifications states, and the relative ratio of H3K4me3 and H3K27me3 varied broadly. In contrast, H3.3 incorporation into HK and CBL genes was almost exclusively and preferentially associated with H3K4me3 and H3K27me3, respectively. In E14.5 hindlimb tissue where SKM genes are committed and expressed, H3.3 in SKM genes was more associated with H3K4me3 than H3K27me3, which is consistent with H3.3 and H3K4me3 association with a transcriptionally active state (50,51). In contrast, in liver tissue, where SKM genes are not committed or expressed, H3.3 in SKM genes was more associated with H3K27me3. H3.3 in HK genes remained associated with H3K4me3 in both the hindlimb and liver. In CBL genes, H3.3 was more associated with H3K27me3. Re-ChIP assay supported bivalent modification at SKM genes in E10.5 trunk (Supplementary Figure S7). These profiles of H3.3 and modifications in different tissues suggest that the amount of incorporated H3.3 is correlated with a quantitative balance between H3K4me3 and H3K27me3 modifications in lineage-specific genes (Figure 7).

## DISCUSSION

In a previous study, we showed that the histone H3.3 variant is incorporated into SKM genes prior to muscle cell differentiation (13). Here, to determine the significance of H3.3 on lineage-specific genes, we forced H3.1 incorporation into C2C12 cells by overexpressing GFP-H3.1 independently of the cell cycle. The replacement of H3.3 with GFP-H3.1 in lineage-specific genes altered the bivalent modification state to increase the H3K27me3 level, probably through an enhanced recruitment of Ezh2 and resulted in inhibiting lineage-specific gene expression and differentiation. Alteration of H3.3 levels by knockdown in C2C12 cells and ectopic MyoD expression in fibroblasts also revealed that both H3K4me3 and H3K27me3 are associated with H3.3 incorporation. A correlation of H3.3 and bivalent modification in lineage-specific genes was also observed in mouse embryos. Together, these findings suggest that proper selection of histone H3 variants regulates the epigenetic state and acts as the lineage potential, with H3.3 possibly functioning to maintain the balance of bivalency between H3K4me3 and H3K27me3 before differentiation.

In bivalent mono-nucleosomes, H3K4me3 and H3K27me3 are present in different H3 tails (52). The formation and maintenance of ‘bivalent genes’ may be carried out by sequential, repetitive and simultaneous de-methylation at one tail and methylation at another, thus alternating H3K4me3 and H3K27me3 modifiers antagonistically (53–55). H3.1 incorporation in lineage-specific genes can alter the balance of the two modifications, which in turn reduces the lineage potential. This consanguineous relationship between H3 variants and histone modification is consistent with biochemical data (8–10) and the H3 barcode hypothesis in which incorporation of appropriate histone H3 variants involves the formation of more stable and selective histone modifications (56).

Among histone H3.3 chaperones, Hira KD affected the myogenic gene expression, including MyoD. This result is probably due to the global suppression of H3.3 incorporation, consistent with its general role in H3.3 chromatin assembly (43). In contrast, Daxx KD had little effect on myogenic gene expression, which is in agreement with its role in H3.3 incorporation into pericentric heterochromatin and teromeres with Atrx (6,57). Finally, Chd2 KD also diminished H3.3 and bivalent modification in SKM genes.

We showed that replacement of H3.3 with H3.1 inhibited the expression of SKM genes prior to transcription, but had no effect on HK genes, including no modifications to H3K4me3, H3K27me3 or even transcription. These results suggested that once genes are actively transcribed, the choice of the histone variant around the promoter regions has little effect on the histone modification or transcription state.

Previously, H3.3 knockdown in mouse ES cells caused a decrease in H3K27me3 levels but no change in H3K4me3 levels (34). In contrast, we observed decreased in both marks. The observation that both H3K4me3 and H3K27me3 levels decreased with depletion of H3.3 incorporation might be explained by a loss of nucleosomes in active regions owing to the high turnover of H3.3 nucleosome at promoter region (37–39). Additionally, the difference between mouse ES cells and our C2C12 cells is likely to be caused by the difference in pluripotency of mouse ES cells and unipotency of C2C12 cells. In ES cells, active and inactive regions on the chromosome are randomly distributed, but in differentiated cells they are clearly compartmentalized in the nuclei (58). In addition, developmental genes with the bivalent state are only slightly expressed in mouse ES cells. These different properties might account for why H3.3 knockdown in mouse ES cells did not change H3K4me3 levels. Our data suggest that appropriate H3.3 incorporation might be required for the future gene expression potential. The replacement of H3.3 to H3.1 might reflect the loss of the gene expression potential upon specific differentiation. Bivalent modification, which is involved in the pre-activation state of the promoter, is often observed in lineage-regulatory genes (2–4,59). Thus, formation of the bivalent modification upon H3.3 incorporation might further enhance the poised state, which is a preparatory step that quickly switches the expression of genes on or off. Therefore, incorporation of H3.3, in association with specific modifications of the tail and specific transcription factors, appears to contribute to the establishment of lineage potential. Further, our results demonstrate that H3.1 incorporation can alter the poised state and lineage potential by ectopic expression. Such active replacement of H3.3 with H3.1 may also regulate the chromatin state to gene repression during differentiation. Thus, the formation of the bivariant state may act an intermediate state of poised genes that promotes the conversion of the bivalent modification to repressive modification after multi-potent stem cells commit to a specific cell fate.

## ACCESSION NUMBERS

ChIP-seq and RNA-seq data were deposited into the DNA Data Bank of Japan database (accession numbers DRA000992-0001002, DRA001256 and DRA000457).

## SUPPLEMENTARY DATA

Supplementary Data are available at NAR Online.

SUPPLEMENTARY DATA
