# Supplementary material for: Incorporation of histone H3.1 suppresses the lineage potential of skeletal muscle
Source: Nucleic Acids Res. 2014 Dec 24;43(2):775–86. doi: 10.1093/nar/gku1346 (PMC4333396; doi:10.1093/nar/gku1346)
Supplement: SUPPLEMENTARY DATA [file supp_43_2_775__index.html]

Incorporation of histone H3.1 suppresses the lineage potential of skeletal muscle — SUPPLEMENTARY DATA 

# Incorporation of histone H3.1 suppresses the lineage potential of skeletal muscle

## SUPPLEMENTARY DATA

**Files in this Data Supplement:**

- SUPPLEMENTARY DATA
- SUPPLEMENTARY DATA
- SUPPLEMENTARY DATA
- SUPPLEMENTARY DATA
- SUPPLEMENTARY DATA
- SUPPLEMENTARY DATA
- SUPPLEMENTARY DATA
- SUPPLEMENTARY DATA
- SUPPLEMENTARY DATA
