# Supplementary material for: Incorporation of histone H3.1 suppresses the lineage potential of skeletal muscle
Source: Nucleic Acids Res. 2014 Dec 24;43(2):775–86. doi: 10.1093/nar/gku1346 (PMC4333396; doi:10.1093/nar/gku1346)
Supplement: SUPPLEMENTARY DATA [file supp_gku1346_nar-03342-x-2014-File011.pdf]

Table S2. ChIP primer list

Acta1 -81 -110: 5′-TGTTGCTGCCCTTCCCAAGCCATATTTGGG-3′
Acta1 -236 -265: 5′-GCAGACAGCTGGGGATACTCTCCATATACG-3′

Ckm -221 -192: 5′-TAGTTTAGGAACCAGTGAGCAAGTCAGCCC-3′
Ckm -114 -85: 5′-CCTGAGAGTAGATGAGCTTTCAGCTCGTTG-3′

Eef1a1 -186 -213: 5′-CGGGTACCGGGATTATGGGTTATGAGAA-3′
Eef1a1 -317 -343: 5′-TAGGGATAGATTCTGGCCGCCCTCTTG-3′

Neurod6 -19 +12: 5′-TTTTTCCCTATCAGTCTAACCTCCTGTGTTGA-3′
Neurod6 -174 -145: 5′-AAAAGTGACATTGATGCCAACTGCCAGAGC-3′

Tbx15 -773 -744: 5’-CCAGATCTGCATCTGAACTGAGTATGATAG-3′
Tbx15 -680 -651: 5’-GTCAAAATCTGATCTTCGCCCTTATAGACT-3′

Hspb2 -277 -250: 5’-AGTTGTACATTCCACATCACCCTTTGTC-3′
Hspb2 -401 -372: 5’-CAGAAGACACTGACCTTACAAAGACAGAAA-3′

Anapc5 -206 -179: 5’-GATTTTGATCAGAGACAGCGTGAACTAC-3′
Anapc5 -320 -291: 5’-TATACACATATACAGGCGAGGTGTCTTGTT-3′

Hdgf -593 -564: 5’-CCTTAGAATATAACCCAGGCCAAATACAGT-3′
Hdgf -490 -461: 5’-GGACTATCACCAATTACCCTCACTAGAATA-3′

Calb1 -807 -778: 5’-CGGATATTTTTCCCACCTCTAATGATTTCC-3′
Calb1 -692 -663: 5’-GTAGAAACTGAAAGAAATGTCACCAGGCTA-3′

Lhx5 -283 -255: 5’-ATTTGTGATGGGAGAGAAAAAGAACCCTG-3′
Lhx5 -180 -151: 5’-CAAGACATCAGTATTGCCGAGTATTTGCTA-3′
