# Supplementary material for: Incorporation of histone H3.1 suppresses the lineage potential of skeletal muscle
Source: Nucleic Acids Res. 2014 Dec 24;43(2):775–86. doi: 10.1093/nar/gku1346 (PMC4333396; doi:10.1093/nar/gku1346)
Supplement: SUPPLEMENTARY DATA [file supp_gku1346_nar-03342-x-2014-File009.doc]

Supplementary Materials

**Supplementary Figures Legend**

Supplementary Figures S1-S7

**Supplementary Figures**

Supplementary Figures S1-S7

**Supplementary Tables Legend**

Supplementary Tables S1-S8

**Supplementary Tables**

Supplementary Tables S1-S8

**Supplementary Figure Legends**

**Supplementary Figure S1. GFP-tagged H3.1 variant under forced expression do not affect endogenous histone expression.** (A) Proportions of GFP-positive cells were determined by flow cytometry after Dox addition. (B) Forced expression of GFP-tagged H3 variants did not affect endogenous histone expression. Immunoblotting was performed to detect GFP, histones, and other indicated proteins. (C) Stable incorporation of GFP-H3.1 and GFP-H3.3 into chromatin and forced expression of GFP-H3 variants did not affect endogenous histone expression. Nuclear-soluble and chromatin fractions purified with hydroxyapatite (HAP) were prepared as described in the Supplemental Experimental Procedures and visualized by silver staining. * denotes GFP-H3 variants and ** indicates the histone octamer component (H2A, H2B, H3, and H4) (D) Stable incorporation of GFP-H3.1 and GFP-H3.3 into chromatin of C2C12 cells. The mobility of GFP-H3.1 and GFP-H3.3 were analyzed by fluorescence recovery after photobleaching half of the nucleus. Examples and recovery curves of GFP-H3.1 and GFP-H3.3 are shown. Relative ﬂuorescence intensities are indicated as means ± standard deviation. (GFP-H3.1, n = 10; GFP-H3.3, n = 9). The slow fluorescence recovery and decay in photobleached and unbleached areas, respectively, indicate stable chromatin incorporation of GFP-H3.1 and GFP-H3.3. Bars = 10 μm. (E) GFP expression induction by Dox did not affect SKM gene expression. qPCR was performed to analyze the levels of the denoted mRNAs. Values of differentiated cells expressing GFP with Dox addition were set to 1. Data represent the mean ± standard deviation of three independent experiments. *Eef1a1* values served as a control.

**Supplementary Figure S2. H3.3 distribution in WT cells and GFP-H3.1 variant expressing cells and nucleosomes purified by hydroxyapatite were found to be mono-, di-, and tri- nucleosomes.** (A) H3.3 incorporation was greater in HK genes than in SKM genes at the gene body. The aggregation plots show H3.3 enrichment as the input-subtracted signal tallied for SKM genes (red), HK genes (green), and silent genes (blue) (Harada et al., 2012). Along the horizontal axis, numbers indicate the number of kb upstream (< 0) or downstream (> 0) the TSS, and gene length was defined as 1 (B) Nucleosome size was determined by 1.5% agarose gel electrophoresis after digestion with MNase.

**Supplementary Figure S3. GFP-tagged histone variants are incorporated into nucleosomes and undergo histone modification.** (A) Immunoprecipitation of H3K4me3 (left panel) or H3K27me3 (right panel) from nucleosomes extracted from GFP-H3.1 and GFP-H3.3 expressing cells. IgG was used as the control. (B) HAP-IP was performed using GFP-H3.1 and GFP-H3.3 expressing C2C12 myoblast nucleosome extracts with GFP-specific antibodies or IgG controls. (C) GFP-H3.1 and GFP-H3.3 variant underwent histone modification in chromatin. Immunoblotting was performed from nucleosomes extracted from GFP-H3.1 and GFP-H3.3 expressing cells and WT cells.

**Supplementary Figure S4. Regular ChIP data for Chd2KD and MyoDKD cells.** (A) Western blots evaluating H3.3, Daxx and Hira proteins knockdown and the expression of other proteins as indicated. (B) The transcription of skeletal muscle marker genes was suppressed in C2C12 cells expressing siRNAs targeting *H3.3* (*H3f3a* and *H3f3b), Daxx,* and *Hira*. mRNA levels were analyzed by qPCR; data represent the means of three independent experiments ± standard deviation. (C) ChIP assays for H3K4me3 (left panel) and H3K27me3 (right panel) were performed as described in Fig. 4A at gene promoters for SKM, HK, and silent genes in proliferating Chd2^KD^- (Chd2^miR3139^) and Chd2^WT^-expressing C2C12 cells that were subjected to the differentiation protocol. (D) ChIP assays were performed as described in (C) using C2C12 myoblasts and differentiated cells treated with either control siRNA or MyoD siRNA. (E) The myogenic model systems based on cultured cells. H3.3 incorporation into myogenic genes can induce both H3K4me3 and H3K27me3 to establish lineage potential, while H3.1/H3.2 is preferentially associated with H3K27me3 for gene silencing. **P* < 0.05, ***P* < 0.01.

**Figure S5. GFP-tagged H3 variants with swapped N-tail specific amino acids (H3.1 A31 and H3.3 S31) were incorporated into chromatin.** (A) Stable incorporation of GFP-H3.1 A31S and GFP-H3.3 S31A into chromatin. Immunoblotting was performed from nucleosomes extracted from GFP-H3.1 A31S and GFP-H3.3 S31A expressing cells. (B) Forced expression of GFP-tagged H3 variants did not affect endogenous histone H3 expression. Immunoblotting was performed to detect GFP. Hsp90 and H3 were used as the loading control.

**Supplementary Figure S6. H3.3 and bivalent modification are altered in the vicinity of genes, but not in the whole genome of the mouse embryo.** (A) Pearson’s correlation matrix for ChIP-Seq data. Correlations were calculated from the ChIP-seq signal intensity of 2-kb bins across the entire mouse genome. Mean correlation values for each ChIP-seq data set are indicated in the corresponding blocks (yellow squares). Each ChIP antibody sample was tested by three independent biological experiments. (B) H3.3 was incorporated at E10.5 in trunk tissue before the expression of SKM genes. The aggregation plot shows H3.3 enrichment as the input-subtracted signal tallied for SKM genes (red), HK genes (green), and silent genes (blue) (13). Along the horizontal axis, numbers indicate the number of kb upstream (< 0) or downstream (> 0) the TSS, and gene length was defined as 1. (C) H3.3 incorporation and bivalent modifications are present at E10.5 around genes typically expressed at E14.5. Hindlimb-specific genes (upper panel) and forelimb-specific genes (lower panel) were analyzed at E14.5 (30). Images were generated with Integrated Genome Viewer (http://www.broadinstitute.org/igv/). The vertical axis shows the normalized signal intensity of each genome locus averaged over three independent ChIP-seq datasets.

**Supplementary Figure S7. Re-ChIP assay results for data shown in figure 6B.** H3K4me3 and H3K27me3 co-modification at SKM genes is shown by re-ChIP. Data for 1st antibodies against H3K4me3 and H3K27me3 and 2nd antibodies against H3K4me3, H3K27me3, and H3.3 are indicated. % input was defined as the ratio of amplification of the PCR product relative to 10% of input genomic DNA normalized to the 1st antibody. Data represent the mean of three independent experiments.


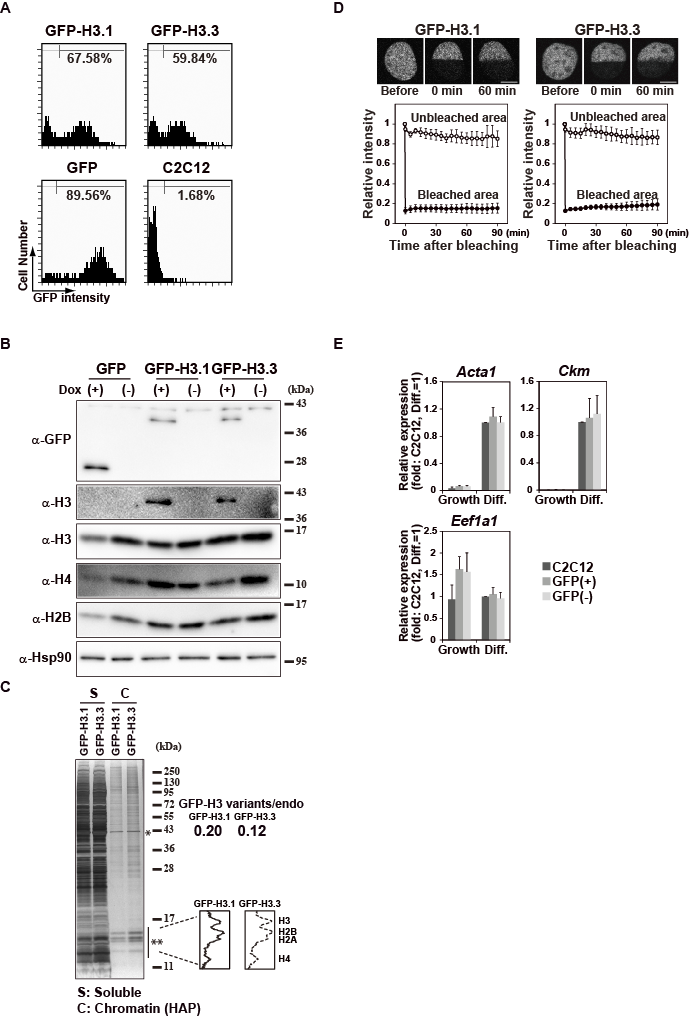


**Supplementary Figure S1**


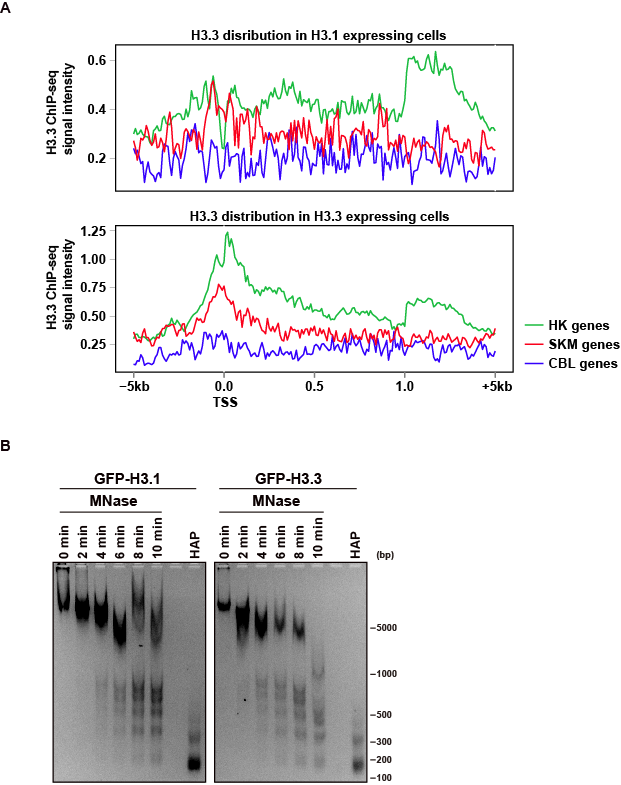


**Supplementary Figure S2**


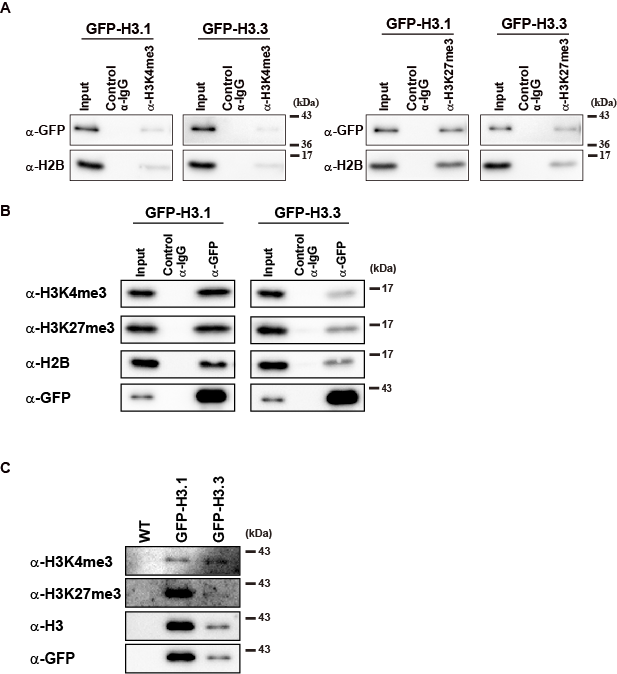


**Supplementary Figure S3**


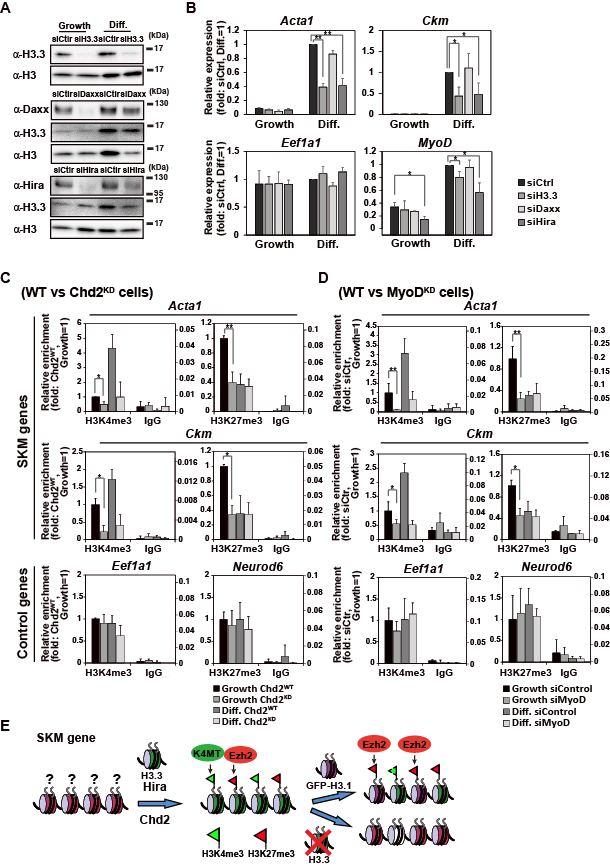


**Supplementary Figure S4**

**
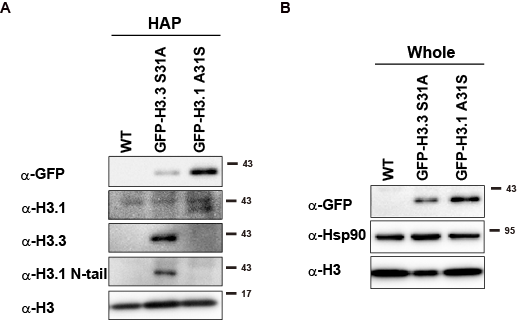
**

**Supplementary Figure S5**

**
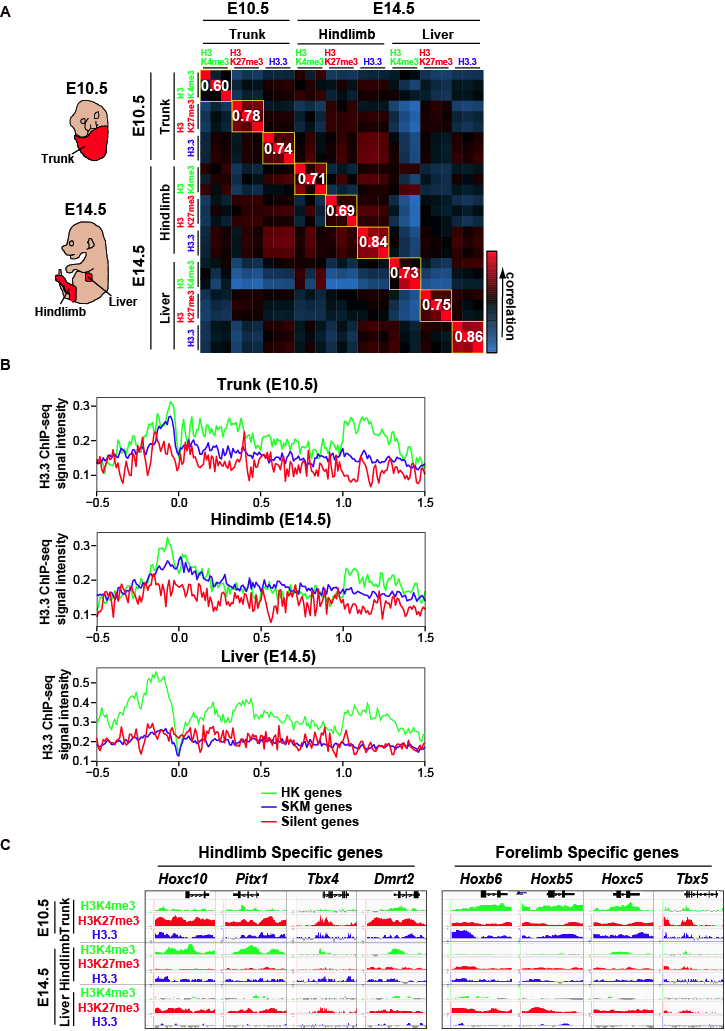
**

**Supplementary Figure S6**


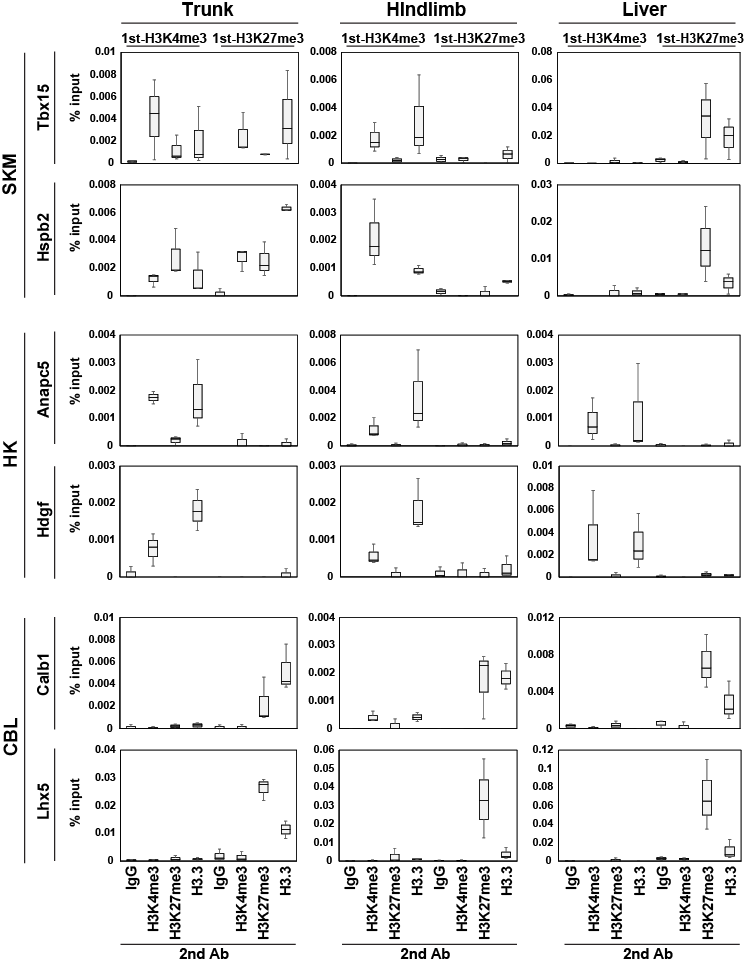


**Supplementary Figure S7**

**Supplementary Table Legends**

**Supplementary Table S1. Quantitative RT-PCR primers.**

**Supplementary Table S2. ChIP primers.**

**Supplementary Table S3. Unique Tag numbers used for ChIP-Seq data analysis.**

**Supplementary Table S4. ChIP-seq signal intensities within 2kb of the TSS in GFP-H3 histone variant expressing C2C12 cells and WT cells.**

List of genes and H3.3, GFP, H3K4me3 and H3K27me3 ChIP-seq signal intensities of the TSS ± 2 kb in GFP-H3 histone variant-expressing C2C12 cells. Sheet #1, WT cells; Sheet #2, GFP-H3.1 expressing cell; Sheet #3, GFP-H3.3 expressing cells.

**Supplementary Table S5. Gene list for Figure 2A and 2B.**

List of genes, GFP enrichment, H3.3 enrichment, and FPKM in C2C12 cells at the growth state and differentiated state (using RNA-Seq). Sheet #1, SKM genes in WT cells; Sheet #2, HK genes in WT cells; Sheet #3, CBL genes in WT cells; Sheet #4, SKM genes in GFP-H3.1 expressing cells; Sheet #5, HK genes in GFP-H3.1 expressing cells; Sheet #6, CBL genes in GFP-H3.1 expressing cells; Sheet #7, SKM genes in GFP-H3.3 expressing cells; Sheet #8, HK genes in GFP-H3.3 expressing cells; Sheet #9, CBL genes in GFP-H3.3 expressing cells.

**Supplementary Table S6. SKM gene list for Figure 6B.**

List of genes, phi (
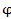
), H3.3 enrichment, and FPKM in C2C12 cells at the growth state and differentiated state (using RNA-Seq). Sheet #1, trunk at embryonic day 10.5; Sheet #2, hindlimb ~~in~~ at embryonic day 14.5; Sheet #3, liver at embryonic day 14.5).

**Supplementary Table S7. HK gene list for Figure 6B.**

List of genes, phi (
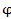
), H3.3 enrichment, and FPKM in C2C12 cells at the growth state and differentiated state (using RNA-Seq). Sheet #1, trunk at embryonic day 10.5; Sheet #2, hindlimb at embryonic day 14.5; Sheet #3, liver at embryonic day 14.5).

**Supplementary Table S8. CBL gene list for Figure 6B.**

List of genes, phi (
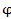
), H3.3 enrichment, and FPKM in C2C12 cells at growth state and FPKM in C2C12 cells at differentiated state (using RNA-Seq). Sheet #1, trunk at embryonic day 10.5; Sheet #2, hindlimb at embryonic day 14.5; Sheet #3, liver at embryonic day 14.5).
