# Supplementary material for: Incorporation of histone H3.1 suppresses the lineage potential of skeletal muscle
Source: Nucleic Acids Res. 2014 Dec 24;43(2):775–86. doi: 10.1093/nar/gku1346 (PMC4333396; doi:10.1093/nar/gku1346)
Supplement: SUPPLEMENTARY DATA [file supp_gku1346_nar-03342-x-2014-File010.doc]

**Table S1. Quantitative RT-PCR primer list**

Acta1:

5’-TTGTGCACCGCAAATGCTTCTAGG-3’

5’-ATGTACACGTCAAAAACAGGCGCC-3’

Ckm:

5’-AAGTCCAATCATTGGGCTCTGTCC-3’

5’-ACGGACTTTTATTTAAGGCAGGGC-3’

Gapdh:

5’-GACTTCAACAGCAACTCCCACTCT-3’

5’-GGTTTCTTACTCCTTGGAGGCCAT-3’

Eef1a1:

5’-CTCTGACTACCCTCCACTTGGTCG-3’

5’-ATTAAGACTGGGGTGGCAGGTGTT-3’

MyoD:

5’-AAATGACACTCTTCCCAACTGTCC-3’

5’-AGGACTATGTCCTTTCTTTGGGGC-3’
