# Supplementary material for: Incorporation of histone H3.1 suppresses the lineage potential of skeletal muscle
Source: Nucleic Acids Res. 2014 Dec 24;43(2):775–86. doi: 10.1093/nar/gku1346 (PMC4333396; doi:10.1093/nar/gku1346)
Supplement: SUPPLEMENTARY DATA [file supp_gku1346_nar-03342-x-2014-File012.doc]

**Table S3. Unique Tag number used for ChIP-Seq data analysis.**

| Library | | Unique tag numbers |
| --- | --- | --- |
| Trunk-lot1 | Input | 11,096,884 |
|  | H3.3 | 7,055,278 |
|  | Input | 18,066,746 |
|  | H3K4me3 | 1,239,481 |
|  | Input | 18,066,746 |
|  | H3K27me3 | 7,136,359 |
| Trunk-lot2 | Input | 18,067,184 |
|  | H3.3 | 9,362,822 |
|  | Input | 30,741,941 |
|  | H3K4me3 | 4,368,529 |
|  | Input | 30,741,941 |
|  | H3K27me3 | 6,278,702 |
| Trunk-lot3 | Input | 19,294,122 |
|  | H3.3 | 9,820,602 |
|  | Input | 19,294,122 |
|  | H3K4me3 | 3,501,683 |
|  | Input | 19,294,122 |
|  | H3K27me3 | 6,012,634 |
| Hindlimb-lot1 | Input | 8,760,088 |
|  | H3.3 | 16,989,581 |
|  | Input | 15,392,028 |
|  | H3K4me3 | 5,004,410 |
|  | Input | 15,392,028 |
|  | H3K27me3 | 4,866,839 |
| Hindlimb-lot2 | Input | 14,201,738 |
|  | H3.3 | 19,535,836 |
|  | Input | 14,201,738 |
|  | H3K4me3 | 10,713,887 |
|  | Input | 14,201,738 |
|  | H3K27me3 | 9,976,809 |
| Hindlimb-lot3 | Input | 15,019,565 |
|  | H3.3 | 14,212,592 |
|  | Input | 7,126,650 |
|  | H3K4me3 | 8,302,518 |
|  | Input | 7,126,650 |
|  | H3K27me3 | 5,074,953 |
| Liver-lot1 | Input | 13,114,050 |
|  | H3.3 | 13,066,320 |
|  | Input | 13,114,050 |
|  | H3K4me3 | 4,312,629 |
|  | Input | 13,114,050 |
|  | H3K27me3 | 3,994,009 |
| Liver-lot2 | Input | 12,931,059 |
|  | H3.3 | 8,959,552 |
|  | Input | 10,104,672 |
|  | H3K4me3 | 1,586,340 |
|  | Input | 10,104,672 |
|  | H3K27me3 | 4,633,767 |
| Liver-lot3 | Input | 15,102,765 |
|  | H3.3 | 12,323,376 |
|  | Input | 15,474,830 |
|  | H3K4me3 | 1,171,904 |
|  | Input | 15,474,830 |
|  | H3K27me3 | 2,682,079 |
| GFP-H3.1 expressing cells | Input | 26,202,245 |
|  | H3.3 | 24,024,892 |
|  | GFP | 34,039,296 |
|  | H3K4me3 | 13,585,187 |
|  | H3K27me3 | 27,176,000 |
| GFP-H3.3 expressing cells | Input | 27,396,125 |
|  | H3.3 | 30,856,338 |
|  | GFP | 30,729,170 |
|  | H3K4me3 | 13,227,790 |
|  | H3K27me3 | 25,310,018 |
| WT cells | Input | 22,793,124 |
|  | H3.3 | 29,116,496 |
|  | Input | 22,533,599 |
|  | H3K4me3 | 3,322,812 |
|  | Input | 22,533,599 |
|  | H3K27me3 | 2,6463,077 |
